# Employing Response Surface Methodology for the Optimization of Ultrasound Assisted Extraction of Lutein and β-Carotene from Spinach

**DOI:** 10.3390/molecules20046611

**Published:** 2015-04-14

**Authors:** Ammar Altemimi, David A. Lightfoot, Mary Kinsel, Dennis G. Watson

**Affiliations:** 1 Department of Plant, Soil and Agricultural Systems, Southern Illinois University, Carbondale, IL 62901, USA; E-Mails: ga4082@siu.edu (D.A.L.); dwatson@siu.edu (D.G.W.); 2 Department of Food Science and Biotechnology, College of Agriculture, University of Basrah, Basrah 61004, Iraq; 3 SIUC Mass Spectrometry Facility, Department of Chemistry and Biochemistry, Southern Illinois University, Carbondale, IL 62901, USA; E-Mail: mkinsel@chem.siu.edu

**Keywords:** lutein, β-carotene, spinach, ultrasound, TLC, densitometry, mass spectrometry

## Abstract

The extraction of lutein and β-carotene from spinach (*Spinacia oleracea* L.) leaves is important to the dietary supplement industry. A Box-Behnken design and response surface methodology (RSM) were used to investigate the effect of process variables on the ultrasound-assisted extraction (UAE) of lutein and β-carotene from spinach. Three independent variables, extraction temperature (°C), extraction power (%) and extraction time (min) were studied. Thin-layer chromatography (TLC) followed by UV visualization and densitometry was used as a simple and rapid method for both identification and quantification of lutein and β-carotene during UAE. Methanol extracts of leaves from spinach and authentic standards of lutein and β-carotene were separated by normal-phase TLC with ethyl acetate-acetone (5:4 (v/v)) as the mobile phase. In this study, the combination of TLC, densitometry, and Box–Behnken with RSM methods were effective for the quantitative analysis of lutein and β-carotene from spinach extracts. The resulting quadratic polynomial models for optimizing lutein and β-carotene from spinach had high coefficients of determination of 0.96 and 0.94, respectively. The optimal UAE settings for output of lutein and β-carotene simultaneously from spinach extracts were an extraction temperature of 40 °C, extraction power of 40% (28 W/cm^3^) and extraction time of 16 min. The identity and purity of each TLC spot was measured using time-of-flight mass spectrometry. Therefore, UAE assisted extraction of carotenes from spinach can provide a source of lutein and β-carotene for the dietary supplement industry.

## 1. Introduction

Medicinal plants have been used throughout human history [1]. Modern researchers have sought to isolate and quantify the natural products underlying the medicinal properties of certain plant parts [2,3]. In addition, the growing interest in functional foods has increased the demand for new food ingredients extracted from natural products [4,5]. Carotenoids are considered one of the most important groups of medicinal natural products. These natural products contribute the yellow to red colors in many flowers, fruits and vegetables. Fruits and vegetables are important sources of two carotenoids important to eye health, lutein and β-carotene. For example, substantial amounts of lutein and β-carotene (40%–60% of daily dietary needs) are found in a single serving of kiwi fruit (*Actinidia* sp.), grapes (*Vitis* sp.), spinach (*Spinacia oleracea* L.), and squashes *Cucurbit* sp. [6]. Spinach is very important for human health and is considered a good source of vitamins A, C, and E. Further, it is very rich in bioactive compounds such as phenolics and carotenoids [7].

Several important health benefits of lutein and β-carotene have been determined [8]. A high concentration of lutein in the *macula lutea* protects against age–related macular degeneration [9]. Lutein and β-carotene are very effective in neutralizing a highly reactive form of oxygen called singlet oxygen [10]. Further, populations with low amounts of β-carotene in their blood often have a higher incidence of heart disease and cancer, particularly lung cancer [4].

Researchers have discovered many advanced methods to isolate and measure the activity of antioxidant compounds such as flavonoids, phenolic acids, tocopherols, carotenoids, and ascorbic acid [11]. A recent review article regarding thin layer chromatography (TLC) analysis of carotenoids in plant and animal samples emphasized the need to study the application of scanning densitometry in quantification of carotenoids and flavonoids [12]. TLC is widely used because it is relatively simple, rapid, inexpensive, and accurate method for chemical identification and can be verified with MS. TLC combined with densitometry and image analysis offers quantitative analysis of medicinal plant components. Densitometry can be used to measure the differences among absorbance or fluorescence signals between a separated zone and the empty plate background across a range of wavelengths [13]. Image analysis methods are used to compare the spot color intensity with the plate color background. The peak area of the test spots are compared with data from calibration standards chromatographed on the same plate [14].

Several techniques have been used for analyzing plant extracts, including TLC [15], gas chromatography-mass spectrometry (GC-MS) [16], and high-performance liquid chromatography (HPLC) [5]. HPLC has been widely used, but TLC-densitometry has two advantages, both of increased sensitivity and the ability to process a large number of samples in a short time [17]. Previously, the use of the TLC-densitometry method had not been reported for quantitative determination of lutein and β-carotene from spinach. Ultrasonic treatment in food processing was evaluated by Chemat *et al.* [18] and was found to be very effective by decreasing the processing time, reducing the cost of extraction, preventing thermal damage, and enhancing food quality. The aim of this research was to optimize ultrasonic-assisted extraction of lutein and β-carotene from spinach. Further, the identities and purity of the natural products in the TLC spots were assayed using matrix-assisted laser desorption/ionization time-off-light mass spectrometry (MALDI-TOF MS) analysis.

## 2. Results and Discussion

### 2.1. Chromatographic Separation and Image Analysis Software

TLC-densitometry coupled with image analysis detection was evaluated for the quantitative determination of induced carotenoid. The method was suitable for rapid quantification of lutein and β-carotene in spinach extracts. It required less time for sample preparation and quantification compared to HPLC. These findings were in reasonable agreement with [19].

### 2.2. Fitting the Models

The results of the lutein and β-carotene extractions as measured by TLC-densitometry for each of the Box-Behnken design variable settings were summarized in Table 1. Multiple regression analysis was completed by fitting the data to the quadratic polynomial model (Equation (3)). The analysis of variance (ANOVA) and regression coefficients for the resulting model were presented in Table 2 and indicate the contribution of the variables to the quadratic model [20]. In general, the lack of fit test for the model describes the variation in the data around the fitted model [21]. If the model does not fit the data well, the value of lack of fit will be significant. In that case proceeding with optimization of the fitted response surface is likely to give misleading results. For both lutein and β-carotene, the lack of fit was not significant (*p* > 0.05), indicating the validity of the response surface results.

### 2.3. Effect of Ultrasonic Parameters on Lutein and β-Carotene Contents of Spinach and Analysis of Response Surfaces

Table 2 shows the analysis of variance of the fitted quadratic polynomial model for lutein and β-carotene content. For lutein content (μg/g), the linear parameter (X_2_), the interaction parameter (X_2_X_3_), and the quadratic parameters $\left({\text{X}}_{1}^{2},{\text{X}}_{2}^{2},{\text{X}}_{3}^{2}\right)$ were significant (*p* <0.05). Similarly for β-carotene content (μg/g), the linear parameter (X_2_), the interaction parameter (X_2_X_3_), and the quadratic parameters $\left({\text{X}}_{1}^{2},{\text{X}}_{2}^{2},{\text{X}}_{3}^{2}\right)$ were significant (*p* < 0.05). The results indicate the models used to fit response variables were adequate to represent the relationship between the response values and the independent variables. The R^2^ of the models for lutein and β-carotene content were 0.96 and 0.94, respectively. Moreover, the coefficients of variation (CV) were 1.58 and 1.53, respectively. A relatively lower value of CV indicates a better reliability of the response model [22].

**Table 1:** Summary of independent variable settings of ultrasonic treatments for Box-Behnken design, amounts of lutein and β-carotene extracted from spinach, and predicted values based on RSM model.

Run	Factor X_1_: Temperature (°C)	Factor X_2_: Power (%)	Factor X_3_: Time (min)	Lutein μg/g		β-Carotene μg/g	
				Actual	Predicted	Actual	Predicted
1	40	30	10	2.08	2.05	3.10	3.07
2	50	50	10	1.92	1.91	2.90	2.88
3	40	70	10	1.82	1.85	2.80	2.82
4	30	50	30	1.88	1.89	2.86	2.88
5	40	50	20	2.04	2.06	3.08	3.10
6	40	70	30	1.97	1.98	2.93	2.96
7	50	50	30	1.84	1.85	2.81	2.78
8	30	30	20	1.87	1.89	2.90	2.90
9	40	50	20	2.07	2.06	3.11	3.10
10	50	70	20	1.83	1.81	2.80	2.80
11	30	50	10	1.88	1.87	2.82	2.85
12	30	70	20	1.85	1.83	2.91	2.86
13	40	50	20	2.09	2.06	3.12	3.10
14	50	30	20	1.87	1.89	2.84	2.89
15	40	50	20	2.03	2.06	3.06	3.10
16	40	30	30	1.92	1.89	2.88	2.86
17	40	50	20	2.09	2.06	3.12	3.10

**Table 2:** Analysis of variance results for the multiple regression to predict lutein and β-carotene.

Source	Degree of Freedom	Sum of Square	Mean Square	f-Value	*p*-Value
lutein					
Model	9	0.1560	0.017	18.40	0.0004
X_1_	1	4.9999	4.9999	0.053	0.8244
X_2_	1	0.0091	0.0091	9.67	0.0171
X_3_	1	0.0010	0.0010	1.07	0.3344
X_1_X_2_	1	0.0001	0.0001	0.11	0.7541
X_1_X_3_	1	0.0016	0.0016	1.70	0.2337
X_2_X_3_	1	0.0240	0.0240	25.50	0.0015
${\text{X}}_{1}^{2}$	1	0.0804	0.0804	85.42	<0.0001
${\text{X}}_{2}^{2}$	1	0.0210	0.0210	22.37	0.0021
${\text{X}}_{3}^{2}$	1	0.0088	0.0088	9.35	0.0184
Lack of fit	3	3.475	0.0011	1.49	0.3461
β-carotene					
Model	9	0.2336	0.026	12.86	0.0014
X_1_	1	0.00245	0.00245	1.21	0.3070
X_2_	1	0.0097	0.0097	4.85	0.0434
X_3_	1	0.00245	0.00245	1.21	0.3070
X_1_X_2_	1	0.0006	0.0006	0.31	0.5952
X_1_X_3_	1	0.004225	0.004225	2.09	0.1912
X_2_X_3_	1	0.030625	0.030625	15.17	0.0059
${\text{X}}_{1}^{2}$	1	0.1047	0.1047	51.91	0.0002
${\text{X}}_{2}^{2}$	1	0.0254	0.0254	12.61	0.0093
${\text{X}}_{3}^{2}$	1	0.0362	0.0362	17.94	0.0039
Lack of fit	3	0.011	3.750	5.21	0.0724

In order to visualize the relationship between the response and experimental levels of the independent variables for the UAE, three-dimensional (3D) surface plots were constructed according to the following quadratic polynomial model equations of coded factors:

(1) $$\text{Lutein }=\;2.064\;-\;0.0024{\text{X}}_{1}-\;0.03375{\text{X}}_{2}-\;0.01125{\text{X}}_{3}-\;0.005{\text{X}}_{1}{\text{X}}_{2}\;-\;0.02{\text{X}}_{1}{\text{X}}_{3}+\;0.0775{\text{X}}_{2}{\text{X}}_{3}-\;0.13825{\text{X}}_{1}^{2}\;-\;0.07075{\text{X}}_{2}^{2}-\;0.04575{\text{X}}_{3}^{2}$$

(2) $$\text{B-carotene }=\;3.098\;-\;0.0175{\text{X}}_{1}-0.035{\text{X}}_{2}-\;0.0175{\text{X}}_{3}-\;0.0125{\text{X}}_{1}{\text{X}}_{2}\;-\;0.0325{\text{X}}_{1}{\text{X}}_{3}+\;0.0875{\text{X}}_{2}{\text{X}}_{3}-\;0.15775{\text{X}}_{1}^{2}-\;0.07775{\text{X}}_{2}^{2}-\;0.09275{\text{X}}_{3}^{2}$$

**Figure 1:**
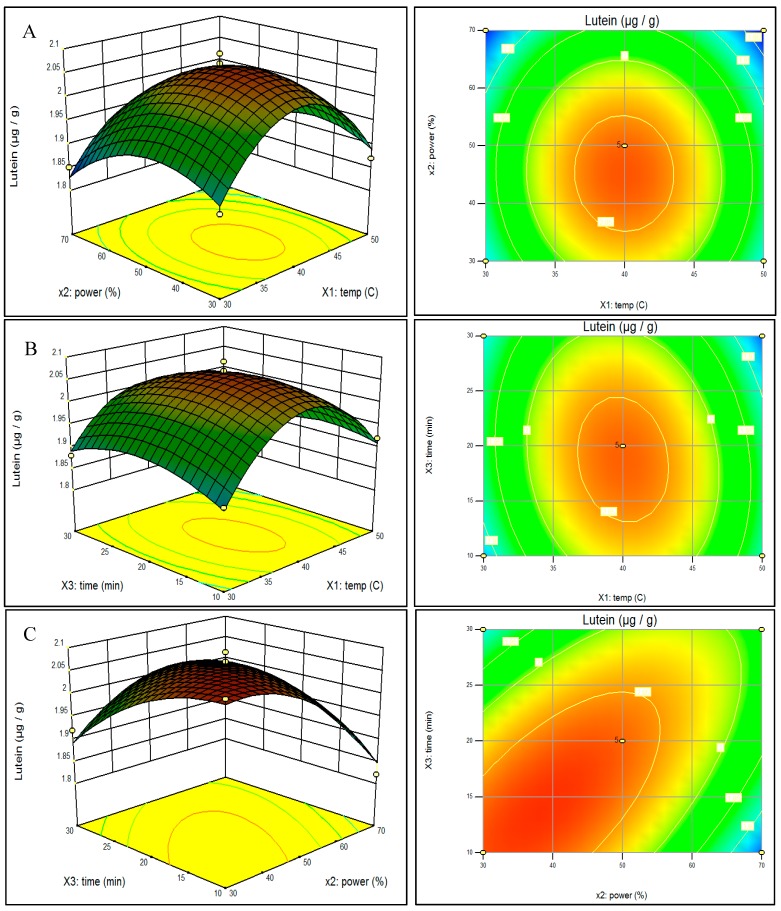
Response surface model plot showing the effects of independent variables on Lutein content: panel **A** temperature and power; panel **B** temperature and time; and panel **C** power and time.

The effect of the variables and their interaction on predicted lutein extraction can be seen in Figure 1. As shown in Figure 1A,B, lutein content was positively correlated with extraction temperature when temperature was lower than about 40 °C. However, lutein was negatively correlated when temperature increased beyond about 40 °C when extraction time was fixed at 20 min (panel A) and when power was set at 50% (panel B). The optimum temperature for maximum lutein extraction was 40.5 °C. These current results were in agreement with Palma and Taylor [23]. It is true that higher temperatures lead to increase in diffusion coefficient and solubility but at the same time the high temperatures may result in degradation of phenolic natural products. Figure 1C with temperature set at 40 °C, showed increased lutein extraction at lower time and power settings. The response surface method took into account the possible interrelationships among the test variables thereby minimizing the number of experiments needed. Therefore, the time and costs of UAE were reduced compared to the conventional optimization method where only one factor is varied at a time while all the others are kept constant, and which ignores the combined interactions between variables [24]. Rebecca *et al.* [25] also used a conventional extraction method to extract carotenoids such as lutein and β-carotene from red capsicum (*Capsicum annuum* L.), yellow capsicum (*C. annuum*), red spinach (*Amaranthus dubius* L.), carrot (*Daucus carota* L.) and broccoli (*Brassica oleracea* L.). Although no comparative studies of conventional and UAE methods with spinach have been reported, conventional methods require more steps in preparation, have a loss of solvents, require more time, and require multiple separation steps. For lutein extraction, the optimum power setting was 38.3% and optimum time was 16.2 min.

**Figure 2:**
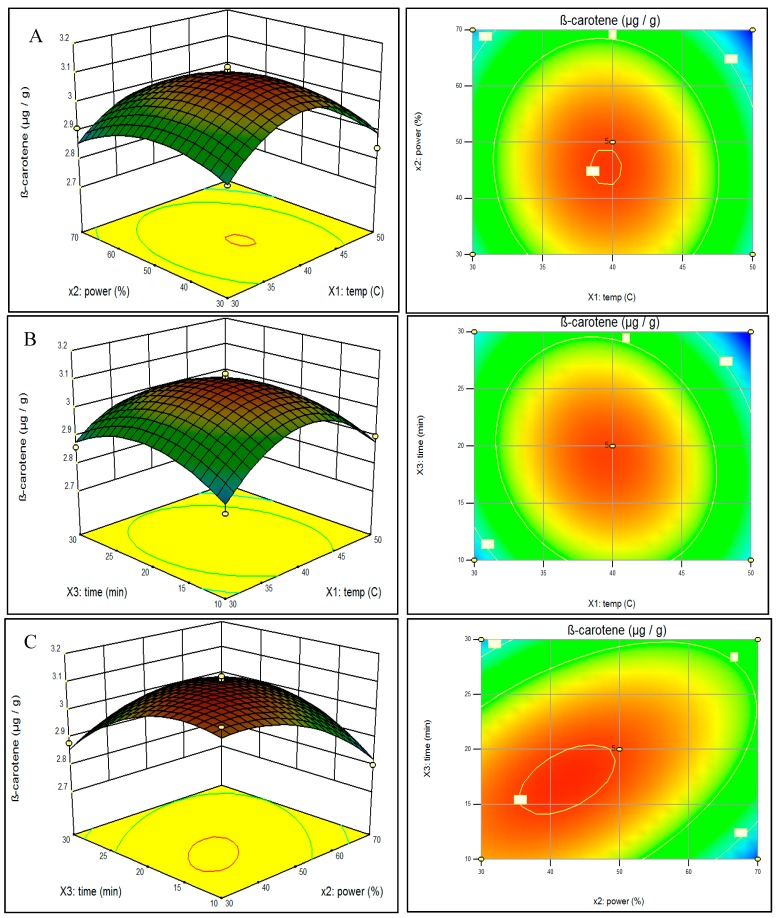
Response surface model plot showing the effects of independent variables on β-carotene content: panel **A** temperature and power; panel **B** temperature and time; and panel **C** power and time.

Figure 2 illustrates the effect of the variables and their interaction on predicted β-carotene extraction. Figure 2A shows the effect of the interaction of extraction temperature and power at a fixed extraction time of 20 min and Figure 2B shows time and temperature with power fixed at the midpoint of 50%. Similar to lutein yield, β-carotene extraction seems to peak near 40 °C temperature. With temperature held at the midpoint of 40 °C (Figure 2C), maximum β-carotene yield is observed when both time and power are below their respective midpoints. The optimized parameter settings for predicted β-carotene output were 39.9 °C, 42.5% power, and 17.3 min.

### 2.4. Optimization and Verification of the Model for Ultrasonic Parameters

Optimum process parameters were determined by simultaneously maximizing lutein and β-carotene extractions. During the optimization stage, the desirability function of the Design-Expert^TM^ software (version 9) statistical software was used to obtain the best compromise of all responses. The predicted optimal conditions for simultaneous ultrasonic extraction were found at 40.1 °C temperature, 41.1% power, and 16.2 min of ultrasonic treatment with results of 2.08 μg/g of lutein and 3.11 μg/g of β-carotene. The UAE process was repeated at near optimum conditions by modifying the extraction temperature of 40.1 °C to 40 °C, and extraction power 41.1% to 40% and extraction time 16.2 min to 16 min. With these settings, total extracted lutein and β-carotene contents were 2.01 ± 0.04 μg/g and 3.07 ± 0.04 μg/g, respectively. Table 3 summarizes the amounts of lutein and β-carotene content under the optimal predicted conditions and actual experimental conditions. There was no significant difference (*p* > 0.05) between the experimental and predicted values. Hence, the models can be used to optimize the process of lutein and β-carotene contents from spinach.

**Table 3:** Predicted and actual experimental values of lutein and β-carotene (μg/g) from spinach extracts under the modified optimal extraction conditions.

Name	Extraction Variables			Lutein (LS)	β-Carotene (BS)
	X_1_ (°C)	X_2_ (%)	X_3_ (min)		
Optimum conditions( predicted)	40.14	41.13	16.21	2.07	3.10
Modified optimal condition (experimental values) *	40	40	16	2.01 ± 0.040	3.07 ± 0.02

* Mean ± standard deviation (n = 3).

**Figure 3:**
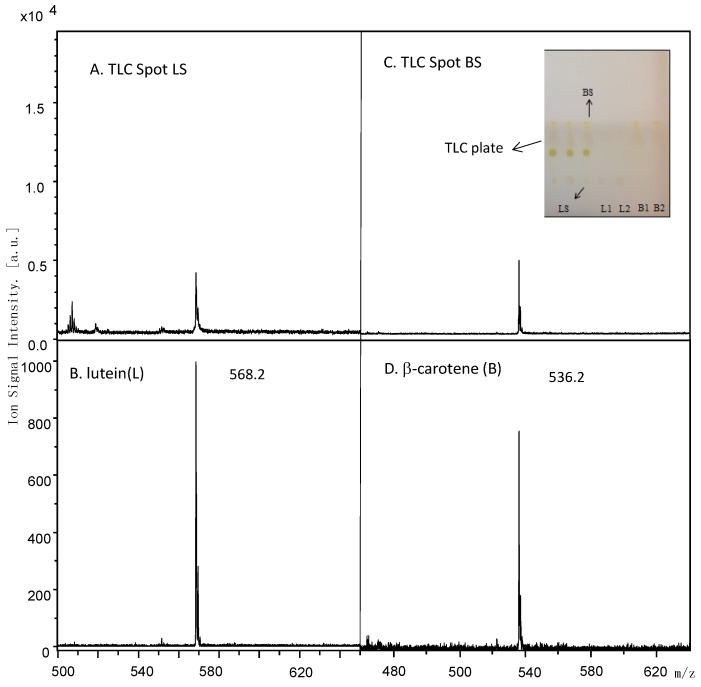
Mass spectra of spinach extract TLC spot LS of experimental condition (panel **A**) and TLC spot BS of experimental condition (panel **C**) excised and compared to lutein (panel **B**) and β-carotene (panel **D**) standards.

### 2.5. MALDI Identification

Figure 3 shows the MALDI mass spectra obtained for TLC Spot LS (Figure 3A) and TLC Spot BS (Figure 3C). Ion signals were observed at *m/z* 568.2 and 536.2. These ion signals were also observed in the MALDI mass spectra obtained from the certified lutein (Figure 3B) and β-carotene (Figure 3D) standards, respectively, and were assigned to the molecular radical cations (M^+.^) of each standard. Thus the lutein and β-carotene identified by TLC-densitometry were confirmed by MALDI mass spectra and appeared as pure as the standards purchased (>98%).

## 3. Experimental Section

### 3.1. Spinach Material

Spinach (cv. ‘Tyee’) was grown according to typical commercial methods for southern Illinois at the Horticulture Research Center of Southern Illinois University (Carbondale, IL, USA). Fresh spinach leaves were harvested from randomly selected plants. The leaves were washed, sliced into small pieces, mixed together, crushed in a blender, sealed in plastic bags, and stored at −18 °C. After five days all samples were freeze-dried.

### 3.2. Ultrasonic–Assisted Extraction (UAE)

An Elmasonic P30 (P30) ultrasonic device with heated water bath (Elma Hans Schmidbauer GMBH, Singen, Germany) set at 37 kHz was used for this study. The P30 had user adjustable controls of heated bath temperatures and power settings. Power could be adjusted as a percentage of full power (30%–100%). The standard ultrasonic mode was used. The manufacturer rated the P30 with an effective power rating of 120 W. The P30 had a proprietary algorithm to adjust power based on the impedance of the system, resulting in the effective power rating. For a specific power setting, samples experienced the same degree of cavitation regardless of the load in the tank. For all treatments, the bath of the P30 contained 1.7 L of water before the treatment containers were added. Ultrasonic power was expressed as W/cm^2^, based on the power setting as a percentage of rated power and the volume of the bath solution.

Although numerous variables my affect a process, identifying and controlling each variable with small contributions is practically impossible, therefore, variables were selected with known major effects [26]. The prior work of Altemimi *et al.* [27] with the same ultrasonic equipment was used as a guide and selected variables were bath temperatures of 30 °C, 40 °C , and 50 °C; power level settings of 30%, 50%, and 70%; and ultrasonic duration of 10 min, 20 min, and 30 min. Based on the manufacturer’s effective power rating, the ultrasonic power for the three power settings inside the extract containers was 21 W/cm^2^, 35 W/cm^2^, and 49 W/cm^2^, respectively. These power settings were independently verified using a calorimetric method.

Crude extracts were prepared as per Altemimi *et al.* [27]. Briefly, ten grams of the lyophilized crushed spinach were placed in a 200 mL glass flask and 100 mL of methanol were added to the flask. Each flask was placed in the P30 and treated. After the samples had been exposed to ultrasound waves, the upper layer was filtered (Whatman no. 1 paper) and placed in a rotary evaporator under vacuum at 40 °C to remove solvent. The spinach preparation, ultrasonic equipment, and treatment process were similar, but a different experimental design was used for the objectives of this study.

### 3.3. Experimental Design

The effects of three independent variables of temperature, power, and time to optimize the extracted amount of lutein and β-carotene was investigated by using a Box–Behnken design for RSM. The coded values of the experimental factors and settings for the experimental design were summarized in Table 4 and the complete list of experiments was included in Table 1. The 17 ultrasonic treatments were completed in random order. The experimental data were analyzed with multiple regression to fit the quadratic polynomial model in Equation (3):

(3) $$Y=b_{0}+\overset{3}{\underset{i=1}{\sum }}b_{i}X_{i}+\overset{3}{\underset{i=1}{\sum }}b_{\text{ii}}{X^{2}}_{i}+\overset{3}{\underset{i\neq j=1}{\sum }}b_{\text{ii}}X_{i}X_{j}$$

where Y is the predicted response; b_0_ is the intercept; b_1_, b_2_ and b_3_ are the linear coefficients of temperature (X_1_), power (X_2_) and time (X_3_), respectively; b_11_, b_22_ and b_33_ are the squared coefficient of temperature of sonication, power and time, respectively; b_12_, b_13_ and b_23_ are the interaction coefficients of temperature of sonication, power and time, respectively. The settings of the independent variables were represented as X_i_ to X_j_.

**Table 4:** Independent variables, symbols and levels used in this Box-Behnken design.

Symbols	Independent Variables	−1	0	1
X_1_	Temperature (°C)	30	40	50
X_2_	Power (%)	30	50	70
X_3_	Time (min)	10	20	30

### 3.4. Thin Layer Chromatography Chemical Screening

The glass TLC plates were 20 cm by 20 cm and pre-coated with silica gel 60 F254 (E. Merck/Millipore, Billerica, MA, USA, 0.2 mm thickness). Three solvents combinations were evaluated for use in the TLC plate to determine the best combination for lutein and β-carotene separation. The solvents were; (1) 5:4 (v/v) ethyl acetate‒acetone; (2) 1:1 (v/v) hexane‒chloroform and (3) 60:3:5 (v/v) benzene‒acetic acid‒water. The TLC plate was placed into oven at 110 °C for 20–30 min to dry completely. Each of the solvents was evaluated by mixing and placing 100 mL into a rectangular chromatography glass tank with ground edges. The glass tank was covered with a glass lid and solvents were allowed to saturate for 30–40 min before use. Two µL of each spinach crude extract was added by syringe to a different TLC plate in a drop shape for identification and spread of the separated compounds according to Harbone [28]. Carotenoids were determined by spraying spots with a 2.54 mM DPPH (diphenylpycrylhydrazyl) methanol solution for derivatization. Spots sprayed with the DPPH were observed as white to yellow bands on a purple background [29]. Images of the TLC plates were analyzed using Quantity One^TM^ software (v6.5; Bio-Rad, Hercules, CA, USA). The compounds in the samples were quantified by comparing density of the peaks and their areas (expressed as intensity per mm^2^) from the samples with those from standard solutions of lutein and β-carotene on the same plate. The best separation was obtained by ethyl acetate: acetone (5:4 (v/v)) and this solvent was used for quantification of lutein and β-carotene.

**Figure 4:**
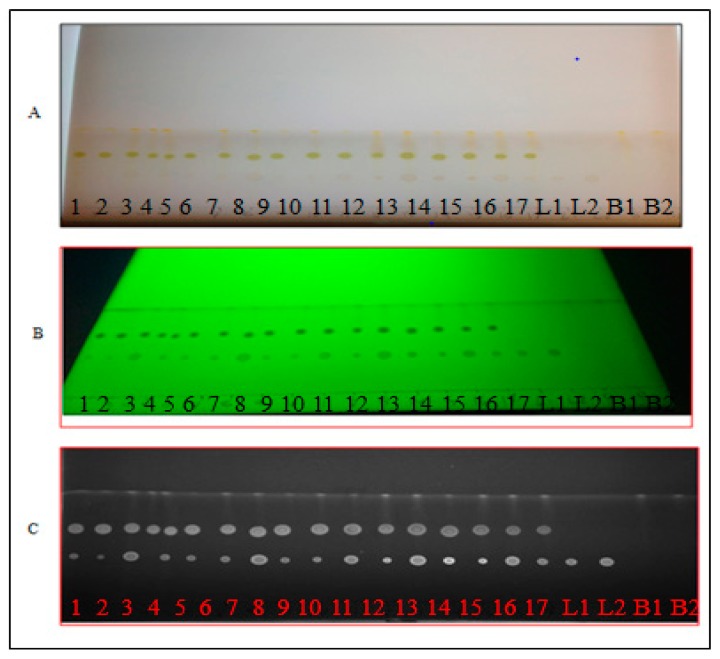
The chromatographic separation of lutein (L) and β-carotene (B): (**A**) real visible light image; (**B**) UV image at 254 nm; and (**C**) grey scale image by Quantity One software. Spots: 1 to 17 for spinach extracts; L1 and L2 for lutein controls; and B1 and B2 for β-carotene controls.

The software evaluated the area of separated spots by comparing the spot color intensity to the color of the TLC plate background. It was essential to chromatograph the standards on the same plates to compensate for slight variations among the different plates (Figure 4). The software generated a calibration curve that allowed the quantitative evaluation of the TLC separation.

### 3.5. Preparation of Calibration Curves for Lutein and β-Carotene

All chemicals used in the experiments were analytical grade reference standard compounds. Lutein (purity 95%) was procured from Indofine Chemical Company (Hillsborough Township, NJ, USA) and β-carotene (purity 98.1%) was purchased from MP Biomedicals LLC (Santa Ana, CA, USA). The stock solution of lutein (100 μg·μL^−1^) was prepared in methanol. Different volumes of the stock solution 2, 4, 6, 8, 10, and 12 μL, were spotted on the TLC plate to obtain concentrations of 200, 400, 600, 800, 1000, and 1200 μg·spot^−1^ of lutein, respectively. The stock solution of β-carotene (80 μg·μL^−1^) was prepared in methanol. Different volumes of the stock solution 2, 4, 6, 8, 10, and 12 μL, were spotted on the TLC plate to obtain concentrations of 160, 320, 480, 690, 800, and 960 μg·spot^−1^ of β-carotene, respectively. These spots of the reference compounds were used to determine the calibration curves for the TLC-densitometry (DOC 2000, Bio-Rad, Hercules, CA, USA). The calibration curves were used by Quantity One^TM^ software to generate accurate quantification of lutein and β-carotene in the experimental samples.

### 3.6. Simultaneous Quantification of Lutein and β-Carotene in the Spinach Extracts

Two µL of each of the 17 spinach extracts were applied on a TLC plate. The plate was developed and scanned as described in the TLC chemical screening process. The peak areas were recorded and the amounts of lutein and β-carotene were calculated using the respective calibration curves.

### 3.7. RSM Model and Validity Testing

Design-Expert^TM^ software (version 9) was used to analyze the experimental results of the response surface design (State-Ease Inc., Minneapolis, MN, USA). A *p*-value less than 0.05 was considered to be significant. Independent variables of extraction temperature, ultrasonic power, and extraction time were simultaneously optimized by using RSM. Subsequently the output for each isolated compound was measured from spinach extracts under the optimum ultrasonic conditions. The ultrasonic experiments using the optimum conditions were replicated three times and the results were compared with the predicted values for validation of the model.

### 3.8. Mass Spectrometric Analysis

The confirmation of each TLC spot identity was achieved using time-of-flight mass spectrometry. Each TLC spot of interest was removed with a scalpel and eluted with methanol and filtered through Xpertek syringe filter (0.24 µm) (P. J. Cobert Associates, Inc., St. Louis, MO, USA) prior to mass spectrometric analysis. The freshly extracted compounds were then prepared for either matrix-assisted laser desorption ionization (MALDI) or laser desorption ionization (LDI). Certified standards of lutein and β-carotene were analyzed in tandem to confirm the identity and purity of each compound.

A 2 μL aliquot of the TLC spot LS methanol extract of experimental condition was mixed with 10 μL hexane. Subsequently, 1 μL of this sample solution was spotted with 1 μL of a saturated solution of the MALDI matrix, a saturated solution of dithranol in hexane on a stainless steel sample plate. TLC Spot BS methanol extract (2 μL) of experimental condition was also diluted in hexane (10 μL) and spotted with dithranol. Both TLC spots LS and BS were allowed to dry at room temperature.

The stainless steel sample plate containing the dried MALDI and LDI samples was inserted into Bruker Daltonics MicroFlexLR time-of-flight mass spectrometer (Billerica, MA, USA). The samples were irradiated with a pulsed nitrogen laser and the positive ion signal was recorded in the mass-to-charge (*m/z*) region of 20 to 1000. Each mass spectrum consisted of an average of 1000 laser shots.

## 4. Conclusions

The results show that TLC-densitometric method & Box–Behnken design can be very powerful techniques for identification and quantitative analysis of carotenoids from spinach extracts. RSM was used to estimate and optimize the experimental variables of extraction temperature (°C), extraction power (%), and extraction time (min). Extraction power, the interaction of power and time, and quadratic of each of the three variables had a significant effect on the response values (lutein and β-carotene). Quadratic models for lutein and β-carotene content were derived with R^2^ = 0.96 and 0.94, for lutein and β-carotene, respectively. Output was optimized for each of lutein and β-carotene separately and simultaneously. The model predictions can be used to optimize lutein and β-carotene extraction from spinach with UAE within the limits of the experimental variables. The modified optimal extraction conditions for measuring lutein and β-carotene simultaneously in spinach extracts were as follows; extraction temperature of 40 °C; extraction power of 40%; and extraction time of 16 min. Under these conditions, the experimental results of total lutein and β-carotene contents were 2.01 ± 0.04 μg/g and 3.07 ± 0.04 μg/g, respectively, which agreed closely with the predicted yield values.

Frozen, dried spinach that has been UAE treated may form a healthful part of the diets of many populations [27]. The extracts produced here could be incorporated into many recipes. Foods high in free beta carotene and lutein could be important components of military rations, where governments have a long term commitment to the healthcare of the service men and women. 

Purified lutein in particular, has a high value in the health supplement industry [30,31,32]. The amounts extracted here could provide a viable alternative to purifying lutein from marigold flowers by column chromatography or re-crystallization methods, which results in 93% purity [30]. Extracts with more than 97% purity are needed for this industry. Spinach could provide a low cost feedstock for the health supplement industry.
